# SfE BES 2026: What’s new in basic science endocrinology?

**DOI:** 10.1530/JME-26-0062

**Published:** 2026-04-24

**Authors:** Gabriela da Silva Xavier

**Affiliations:** ^1^ Department of Metabolism and Systems Science, College of Medicine and Health, University of Birmingham, Birmingham, UK; ^2^ Women’s Metabolic Health Theme, National Institute for Health and Care Research (NIHR), Birmingham Biomedical Research Centre, Birmingham, UK

This year I had the privilege to give the year overview in the session titled ‘What’s new in basic science endocrinology?’ at the Society for Endocrinology’s annual professional conference, SfE BES 2026 (Harrogate, UK, 02–04 March).

SfE BES welcomes a broad church of research and healthcare professionals working within endocrinology, which meant preparing content that was fitting for all. The sheer volume of excellent work across the field that was published in a year made it hard to pick only a few to focus on. A paper is kind of old news by the time it is published – the data could already have been presented and discussed at conferences and/or could have been released as a preprint prior to publication as a manuscript – i.e. the research community with immediate interest in the work would have known of the story for a while and may already have moved on to ‘what next’. This makes the ‘new’ in ‘what’s new’ problematic. However, it is practicable to identify hot topics and emerging themes, as well as areas that are perhaps a bit neglected, based on analysis of publication trends in the past year. Journals do this type of analysis to identify and prepare for topics for which there is potential for more traffic, find topics for special editions, etc. To prepare for the talk, I drew on the analysis of submissions to *Journal of Endocrinology* (JOE) and *Journal of Molecular Endocrinology* (JME), and from other related journals where these were available, and did a systematic review of entries on PubMed.

Looking across the board, the year in publication within endocrinology, and actually also in the general media, was very much dominated by research on the glucagon-like peptide 1 receptor agonists (GLP-1RAs), examples of which are semaglutide (Ozempic), tirzepatide (Mounjaro and Zepbound) and retatrutide. Published research described the mechanisms behind appetite; weight control; improvement in cardiovascular, adipose, liver and kidney function; the potential for use in the treatment of type 1 diabetes; and the physiological impact of discontinuation of treatment with GLP-1RAs. This was a big year for publications on dual and triple agonists, where much discussion was centred on how the combined involvement of hormone receptors could lead to better efficacy for metabolic treatment. This year also saw the publication of a phase 3, multinational, randomised, double-blind trial of orforglipron, an oral non-peptide GLP1-RA that demonstrated enhanced efficacy in weight reduction (1, 2). A small molecule that exhibits biased agonism, orforglipron stimulates GLP-1R-mediated cAMP accumulation selectively over GLP-1R-mediated β-arrestin recruitment (3), thereby enhancing GLP-1R action by reducing the GLP1-R desensitisation pathway (4, 5). Orforglipron is widely cited as a potential transformation in drug delivery, and we are likely to see more research on its mechanism of action in different tissue contexts in the future, much as we have been seeing for the peptide-based GLP-1RAs. As a drug class, there are emerging data on system-wide pleiotropic effects beyond those on metabolism, e.g. potential involvement in mechanisms in substance abuse, biological ageing, and neurological and neurodegenerative disorders, and in the months to come we expect more research data on whether these small molecules do indeed impact such phenomena and, if so, how. One piece of news from the Metabolism Network discussion at SfE BES 2026 was that the rate of weight gain when people come off GLP1-RAs is rapid, just as weight was quick to come off whilst on treatment. The reasons for this and potential workarounds are likely the subject of much discussion in the near future.

I was recently asked by a friend, who is a clinician, whether there was much information on interactions between GLP-1RAs and other medications, e.g. hormone replacement therapy. I looked for an answer to this question whilst conducting my survey for the talk: little is still known and this may, perhaps, be an area of research where some attention may be needed.

Another area of endocrinology that had seen a lot of publications in the last year was endocrine cancer research. One of the papers that has gained attention is one linking tenascin C (a secreted extracellular matrix protein) to potentiation of the Wnt signalling pathway in thyroid cancers, linking modifications of the extracellular matrix to a cellular mechanism that leads to cancer aggression (6). Another paper that links the outside, this time diet, to a pathway for cancer progression was one exploring the link between dietary omega 6 linoleic acid and FABP5-mTORC1 signalling in triple-negative breast cancer disease progression (7). This is significant as linoleic acid, which has been associated with inflammation and cancer, is the most abundant polyunsaturated fatty acid in a Western-style diet, and mTORC1 signalling coordinates cellular growth and nutrient signals. The data presented contributes to the growing body of evidence that suggests that dietary patterns may influence cancer outcomes. Both were elegant and comprehensive studies, with evidence gathered from humans, cell lines and rodent models, using a range of techniques from bioinformatics analysis and *in silico* work to *in vivo* mouse work and wet-lab research, demonstrating an effective research pipeline. The papers gave very detailed descriptions of methodologies with unambiguous reference to the reagents that were used, which would enable replication of the studies. The use of data/animal models of both sexes, as appropriate to the studies, ensured equity in research. The availability of raw data ensures confidence in the findings. These are all practices that we encourage authors that publish with JOE and JME to work towards through our review process. Looking at publication trends and projecting into the future, we will likely see more research probing how the tumour microenvironment and metabolic environment impacts on cancer progression, likely with increased use of patient-derived organoid models.

Looking at submissions and publications at JOE and JME and other related journals, two areas in endocrinology are in the ascendent: endocrine-disrupting chemicals (EDCs) and the application of artificial intelligence (AI) and machine learning in endocrinology research. Acknowledging the rise in submissions involving these emerging topics and the need for efficient crosstalk between the disciplines, in 2025 JOE and JME appointed two new Senior Editors with specialism in these emerging areas – Prof. Jorma Toppari and Dr Eder Zavala – to help us with these publications.

2025 saw the publication of the Endocrine Society Scientific Statement (8) on EDCs and the OECD Report on the State of the Science on EDCs (9), which defined the problem and the methodology for reporting, respectively. Common themes in the publications on EDCs were on the epigenetic and cross-generational impacts of EDCs, neuroendocrine effects, the role of microplastics as EDCs and the use of new approaches and methodologies for the study of the impact of EDCs.

Some of the novel approaches used in EDC research involve the use of AI and machine learning, which is increasingly seen in the literature for the analysis and integration of multi-omics data for the purposes of risk determination, novel diagnostic modalities, disease modelling, defining mechanisms of action/pathways and programme delineation at the cellular, tissue and systems levels. In the use of AI and machine learning for data integration, there is the hope, often expressed in manuscripts, of personalised medicine. Increasing the researcher pool who are literate in both computational techniques and biomedical research would facilitate this venture.

The Co-Editors-in-Chief of JOE and JME have been selecting Editor’s Choice papers every quarter in the past year, as well as the image of the quarter. The papers selected tell a compelling story and with good engagement from the readership of the journals. The Senior Editors are currently voting for their favourites out of these eight for JOE and JME’s Paper of the Year; the results will be announced shortly. The papers shortlisted for 2025 were as follows:

## JOE

Quarter 1 (Q1): ‘Identification of a vimentin-expressing α-cell phenotype in CF and normal pancreas’ (10)

Q2: ‘Maternal sucrose consumption alters steroid levels in the mother, placenta and fetus’ (11)

Q3: ‘The glucocorticoid receptor is affected by its target ZBTB16 in a dissociated manner’ (12)

Q4: ‘Single-cell analysis of oxidative phosphorylation protein expression in pancreatic islets in type 2 diabetes’ (13)

## JME

Q1: ‘The role of mu-opioid receptors in pancreatic islet α-cells’ (14)

Q2: ‘Disruption of thyroid-intrinsic clock aggravates experimental autoimmune thyroiditis’ (15)

Q3: ‘Impact of *Parabacteroides distasonis* colonization on host microbiome, metabolome, immunity, and diabetes onset’ (16)

Q4: ‘*HNF1α*-Q125ter-mediated mitochondrial dysfunction and impaired mitophagy in β-cells’ (17)

I would like to take the opportunity to thank everyone on the editorial team for all their hard work for the journals in the past year.

I think of publications as conversations between members of the research community, and our job at the journals as facilitators of these conversations. In the spirit of inclusiveness and collaboration, we are very pleased that members of the Science Committee will be joining us in some of the new endeavours at JOE and JME in this coming year. In the first of these, the Science Committee’s Choice will replace the Editor’s Choice for quarterly paper selections for the journals. The Paper of the Year will then be voted on from this shortlist (as they were this year) by the Senior Editor team of the journals. Science Committee members will also be giving the ‘What’s New’ talk at the next SfE BES to provide a survey of the field that is more representative of the membership of the SfE and write a review of the year as an accompanying article for the journals.

We are delighted to support two Early Career Editors from the SfE Leadership and Development Awardee cohort. The Senior Editors have also been asked to put forward their idea for an event (e.g. a meeting) that is linked to a special issue for the coming year. The proposals for this year are in; watch this space for an announcement of the selected event. These are pilot projects in our efforts to engage more effectively with the readership of the journals. Do let us know of any suggestions that you may have for how we can do better – we encourage you all to get involved.

## Declaration of interest

The author declares that there is no conflict of interest that could be perceived as prejudicing the impartiality of the work reported.

## Funding

The author received no funding for this work. The work in the author’s lab was funded by the NIHR Birmingham Biomedical Research Center – Women’s Metabolic Health theme and SPARK the Midlands.

## References

[1] Horn DB, Ryan DH, Kis SG, et al. Orforglipron, an oral small-molecule GLP-1 receptor agonist, for the treatment of obesity in people with type 2 diabetes (ATTAIN-2): a phase 3, double-blind, randomised, multicentre, placebo-controlled trial. Lancet 2026 406 2927–2944. (10.1016/s0140-6736(25)02165-8) 41275875

[2] Wharton S, Aronne LJ, Stefanski A, et al. Orforglipron, an oral small-molecule GLP-1 receptor agonist for obesity treatment. N Engl J Med 2025 393 1796–1806. (10.1056/nejmoa2511774) 40960239

[3] Kawai T, Sun B, Yoshino H, et al. Structural basis for GLP-1 receptor activation by LY3502970, an orally active nonpeptide agonist. Proc Natl Acad Sci U S A 2020 117 29959–29967. (10.1073/pnas.2014879117) 33177239 PMC7703558

[4] Marchese A, Paing MM, Temple BR, et al. G protein-coupled receptor sorting to endosomes and lysosomes. Annu Rev Pharmacol Toxicol 2008 48 601–629. (10.1146/annurev.pharmtox.48.113006.094646) 17995450 PMC2869288

[5] Jones B, Buenaventura T, Kanda N, et al. Targeting GLP-1 receptor trafficking to improve agonist efficacy. Nat Commun 2018 9 1602. (10.1038/s41467-018-03941-2) 29686402 PMC5913239

[6] Hartmann HA, Loberg MA, Xu GJ, et al. Tenascin-C potentiates wnt signaling in thyroid cancer. Endocrinology 2025 166 bqaf030. (10.1210/endocr/bqaf030) 39951495 PMC11843548

[7] Koundouros N, Nagiec MJ, Bullen N, et al. Direct sensing of dietary omega-6 linoleic acid through FABP5-mTORC1 signaling. Science 2025 387 eadm9805. (10.1126/science.adm9805) 40080571

[8] Endocrine Society. Endocrine-Disrupting Chemicals: An Endocrine Society Position Statement. Washington, DC, USA: Endocrine Society, 2025. (https://www.endocrine.org/advocacy/position-statements/endocrine-disrupting-chemicals)

[9] OECD. Report on the State of the Science to Address Endocrine Disrupters Under the Globally Harmonised System of Classification and Labelling. Paris, France: Organisation for Economic Co-operation and Development, 2025. (https://www.oecd.org/en/publications/report-on-the-state-of-the-science-to-address-endocrine-disrupters-under-the-globally-harmonised-system-of-classification-and-labelling_9aa7cd81-en.html)

[10] Kattner N, Hang Y, Krentz NAJ, et al. Identification of a vimentin-expressing alpha-cell phenotype in CF and normal pancreas. J Endocrinol 2025 264 e240190. (10.1530/JOE-24-0190) 39836539 PMC11850051

[11] Seib DR, Jung MM & Soma KK. Maternal sucrose consumption alters steroid levels in the mother, placenta and fetus. J Endocrinol 2025 265 e240238. (10.1530/joe-24-0238) 39873388

[12] Galuh S, Faught E, Klaassen I, et al. The glucocorticoid receptor is affected by its target ZBTB16 in a dissociated manner. J Endocrinol 2025 266 e240283. (10.1530/joe-24-0283) 40540650 PMC12231182

[13] Mullins A, Yu X, Smith ALM, et al. Single-cell analysis of oxidative phosphorylation protein expression in pancreatic islets in type 2 diabetes. J Endocrinol 2025 267 e250253. (10.1530/joe-25-0253) 41059747 PMC12910571

[14] Kong C, Castro DC, Lee J, et al. The role of mu-opioid receptors in pancreatic islet α-cells. J Mol Endocrinol 2024 74 e240060. (10.1530/jme-24-0060) 39475846 PMC11875080

[15] Fu J, Gao R, Ye Q, et al. Disruption of thyroid-intrinsic clock aggravates experimental autoimmune thyroiditis. J Mol Endocrinol 2025 74 e250022. (10.1530/jme-25-0022) 39998446

[16] Girdhar K, Randall A, Mine K, et al. Impact of *Parabacteroides distasonis* colonization on host microbiome, metabolome, immunity, and diabetes onset. J Mol Endocrinol 2025 75 e250025. (10.1530/jme-25-0025) 40823966 PMC12400532

[17] Jiang F, Huang J, Chen X, et al. *HNF1α-*Q125ter-mediated mitochondrial dysfunction and impaired mitophagy in β-cells. J Mol Endocrinol 2025 75 e250033. (10.1530/jme-25-0033) 41143604 PMC12910567

